# Blood pressure lowering for prevention of episodic migraine: results of a pilot randomized, placebo-controlled trial of combination blood pressure lowering medication with propranolol

**DOI:** 10.3389/fneur.2025.1630732

**Published:** 2025-09-16

**Authors:** Cheryl Carcel, Faraidoon Haghdoost, Leo Davies, Dennis Cordato, Lyn Griffiths, Grace Balicki, Qiang Li, Ruth Freed, Craig S. Anderson, Alessandro S. Zagami, Candice Delcourt, Anthony Rodgers

**Affiliations:** ^1^The George Institute for Global Health, University of New South Wales, Sydney, NSW, Australia; ^2^Department of Neurology, Royal Prince Alfred Hospital, Sydney, NSW, Australia; ^3^Department of Neurology, Liverpool Hospital,, Liverpool, NSW, Australia; ^4^Centre for Genomics and Personalised Health, Queensland University of Technology, Brisbane, QLD, Australia; ^5^The George Institute China at Peking University Health Science Centre, Beijing, China; ^6^Institute of Neurological Sciences, Prince of Wales Hospital, Sydney, NSW, Australia; ^7^Prince of Wales Clinical School, UNSW, Sydney, NSW, Australia; ^8^Macquarie University, Department of Clinical Medicine, Faculty of Medicine, Health and Human Sciences, Sydney, NSW, Australia

**Keywords:** migraine, antihypertensive treatment, randomized trials, feasibility, tolerability

## Abstract

**Background:**

Beta blockers are effective in migraine prevention. Low-dose combination therapy of blood pressure lowering is likely to lower blood pressure more than a beta-blocker, but there are no data on comparative efficacy of these treatments in migraine prophylaxis.

**Methods:**

A double-blind, randomized, pilot trial in participants with episodic migraine to assess feasibility and tolerability of low-dose triple combination BP lowering (telmisartan 20 mg, amlodipine 2.5 mg and indapamide 1.25 mg) vs. propranolol 160 mg daily vs. matching placebos. The primary outcome was monthly headache days.

**Results:**

Between March 2017 and June 2018, 378 participants were screened and 30 were randomized (8%). The key reasons for ineligibility among screen failures were low blood pressure (86, 26%), unwilling to participate in a drug trial (76, 23%) and not meeting episodic migraine criteria (54, 16%). Among those randomized, mean age was 49 years, 83% female, baseline migraine frequency was 67 h, aura present in 47%, mean baseline BP was 131/87 mmHg. As expected from a small pilot, the confidence intervals for treatment effect estimates were wide: reduction in monthly headache days for the low-dose triple were −0.6 (95% confidence interval [CI] −2.9, 1.8) and for propranolol −1.1 (95% CI −3.8, 1.6) compared to placebo. Tolerability was good, there were no dropouts from adverse events.

**Conclusion:**

As a pilot study, the trial was not powered to detect efficacy; larger trials are required to determine the effect of low-dose triple combination blood pressure lowering on migraine. The medication was safe and well tolerated by participants with migraine; it is likely that study co-design with people with lived experience of migraine will benefit recruitment into the trial.

**Clinical trial registration:**

ACTRN12616000937415, https://anzctr.org.au/.

## Introduction

Beta-blockers are commonly used in migraine prophylaxis, although there is ongoing debate as to their mechanism(s) of action—how much of their benefits are mediated through blood pressure (BP) reduction, their vasodilatory effect, or via central mechanisms (1). However, it is known that numerous other BP lowering drugs are effective in migraine prevention (2). A systematic review showed that headache days were significantly lowered by angiotensin-converting enzyme inhibitors, angiotensin II receptor blockers and calcium channel blockers as well as beta blockers. Standardized mean difference was significantly reduced for all drug classes and was separately significant for numerous specific drugs: clonidine, candesartan, atenolol, bisoprolol, metoprolol, propranolol, timolol, nicardipine and verapamil. Furthermore, in terms of cardiovascular protection, greater BP reduction leads to more benefits—but this has not been evaluated in the context of migraine prophylaxis. Finally, low-dose combinations achieve more BP lowering with better toleralbility than high dose monotherapy, but this intervention has not been tested in migraine (3).

To address these issues, we conducted the Headache Prevention Project (HAPPy), a pilot study for a partial-factorial, double blind, randomized trial to assess the feasibility, safety and effectiveness of BP lowering medications in people with episodic migraine.

## Methods

### Trial design and participants

Participants were recruited from the community and outpatient clinics in New South Wales and Queensland, Australia. Participants were eligible if they were 18 years or older; had 2–14 days of migraine per month over the past 3 months and had an office systolic BP (SBP) of ≥120 mm Hg and a diastolic BP (DBP) of ≥75 mm Hg. These thresholds were selected to minimize the risk of symptomatic hypotension in a population not preselected for hypertension and receiving a fixed-dose triple antihypertensive combination. Exclusion criteria were any contraindication to the medications, abnormal creatinine or electrolytes, and cluster headache or any chronic headaches (headache occurring on more than 15 days per month) (see full list of eligibility criteria in Supplementary Table 1).

The study protocol was approved by the Research Ethics and Governance Office of Sydney Local Health District (Protocol No X15-0410, HREC/15/RPAH/554). Informed consent was obtained from participants.

### Randomization and intervention

**Figure 1:**
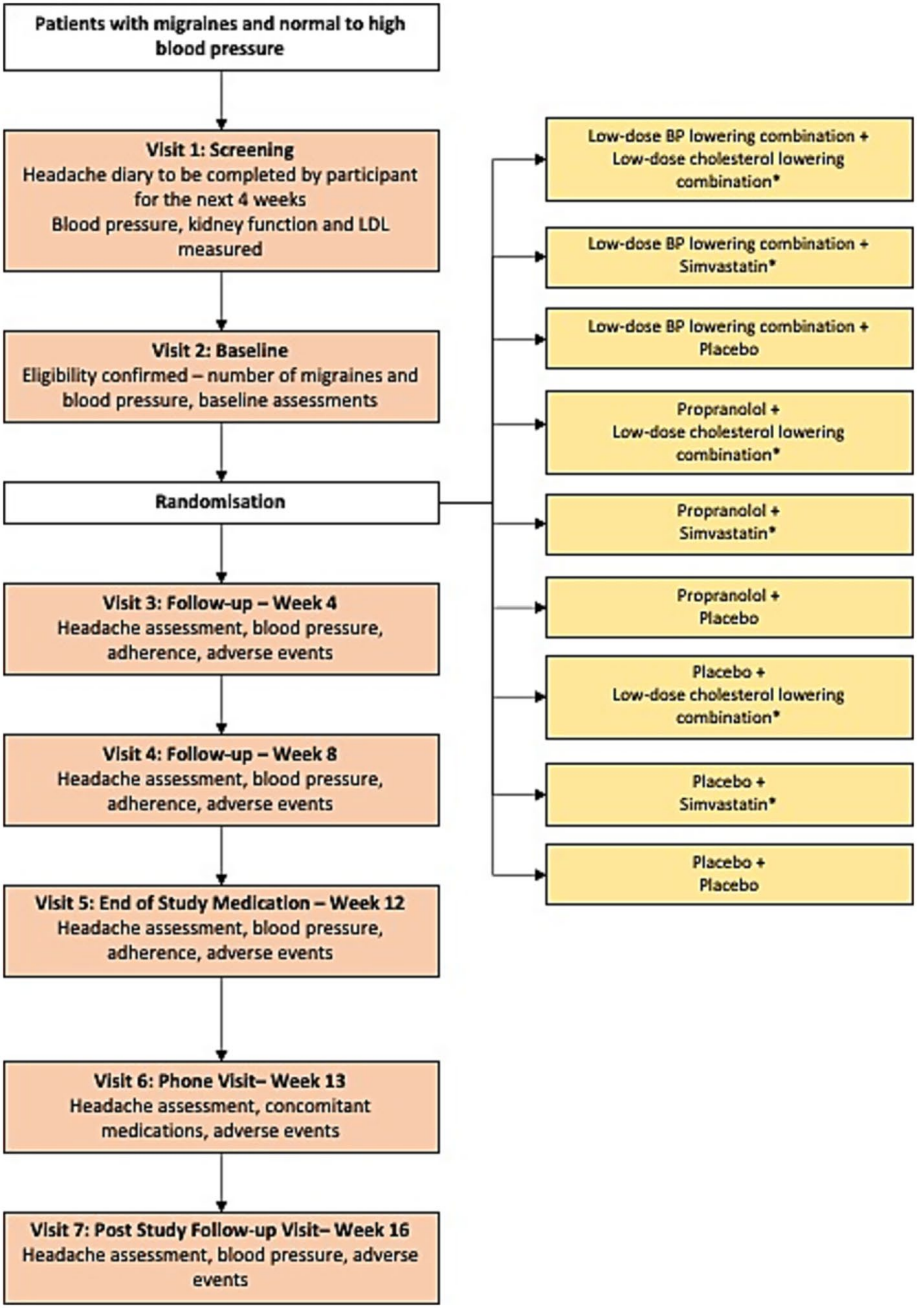
Study design. *The results for the cholesterol lowering arm will be reported separately.

Using a central, computer-based randomization system, participants were randomly assigned to: (1) low-dose BP lowering combination - a single encapsulated pill containing three BP-lowering drugs, at low-dose (telmisartan 20 mg, amlodipine 2.5 mg and indapamide 1.25 mg).; (2) propranolol at a dose of 160 mg/day; or (3) placebo. The placebo capsule appeared identical to, and was of similar weight to, the active drugs. An additional factorial randomization was implemented with participants also allocated to simvastatin 20 mg, low-dose combination cholesterol lowering (rosuvastatin 10 mg plus ezetimibe 10 mg) or placebo (Figure 1). The results for the cholesterol lowering arm will be reported separately. Trial medicines were prepared and packaged at a manufacturing facility licensed with a Certificate of Good Manufacturing Practice by the Therapeutic Goods Administration of Australia.

### Procedures

Recruitment was through general advertisement in hospital clinics, primary care newsletters, health appointment booking sites and a charitable headache organization in Australia. Migraine diagnosis was based on International Classification of Headache Disorders, third edition (ICHD-3) (4). The participants underwent a 4-week screening period, 12 weeks of study medication and another 4 weeks of post study-drug observational period (Figure 1). Visits conducted during the 12 weeks were face-to-face. Post-study follow-up involved a phone call. Biological sex was inquired during the initial study visit. Office BP was measured when the participants were rested in the seated position for 5 min, an appropriate cuff size was selected, and 3 measurements of BP were recorded using an Omron HEM907 monitor or equivalent. The second and third readings were averaged for study analysis. Participants completed paper or electronic based headache diaries at any time a headache occurred after screening. The diary was used to track the number and duration of migraine headache days, acute symptomatic treatment of migraine headache and effectiveness of treatment. All serious adverse events were recorded. Participants and study assessors were blinded to study drug allocation.

### Outcomes

For this pilot trial, testing for trial recruitment and data collection procedures; participant acceptability; and SBP, DBP and LDL differences between the groups were mainly assessed.

The primary outcome was the reduction in mean monthly headache days at 12 weeks when compared to baseline. Secondary outcomes were change from baseline in mean monthly hours with migraine; proportion of subjects with at least a 50% reduction from baseline in monthly headache days; change from baseline in monthly headache medication treatment days; change in SBP and DBP; and adverse events (AEs).

### Statistical analysis

All analyses of study outcomes were conducted according to the principle of intention-to-treat. Mean (standard deviation) and number (%) are reported. The feasibility of the study was assessed as the proportion of patients randomized out of those screened. The pilot was not powered for headache outcomes but rather designed to evaluate feasibility, tolerability, and variance estimates to inform the design of a future, adequately powered efficacy trial. As such, observed treatment effects are considered exploratory and hypothesis-generating. The analyses of continuous outcomes such as change in headache frequency and SBP at 12 weeks were performed using ANCOVA including the treatment arm and baseline values as a covariate by reporting mean change (95% confidence interval). Relative risk (95% confidence interval) was reported for comparison of dichotomous variables such as proportion of participants with at least a 50% reduction in monthly headache. Pre-defined subgroup analyses included headache type, baseline BP, sex and age.

## Results

### Feasibility assessment

**Figure 2:**
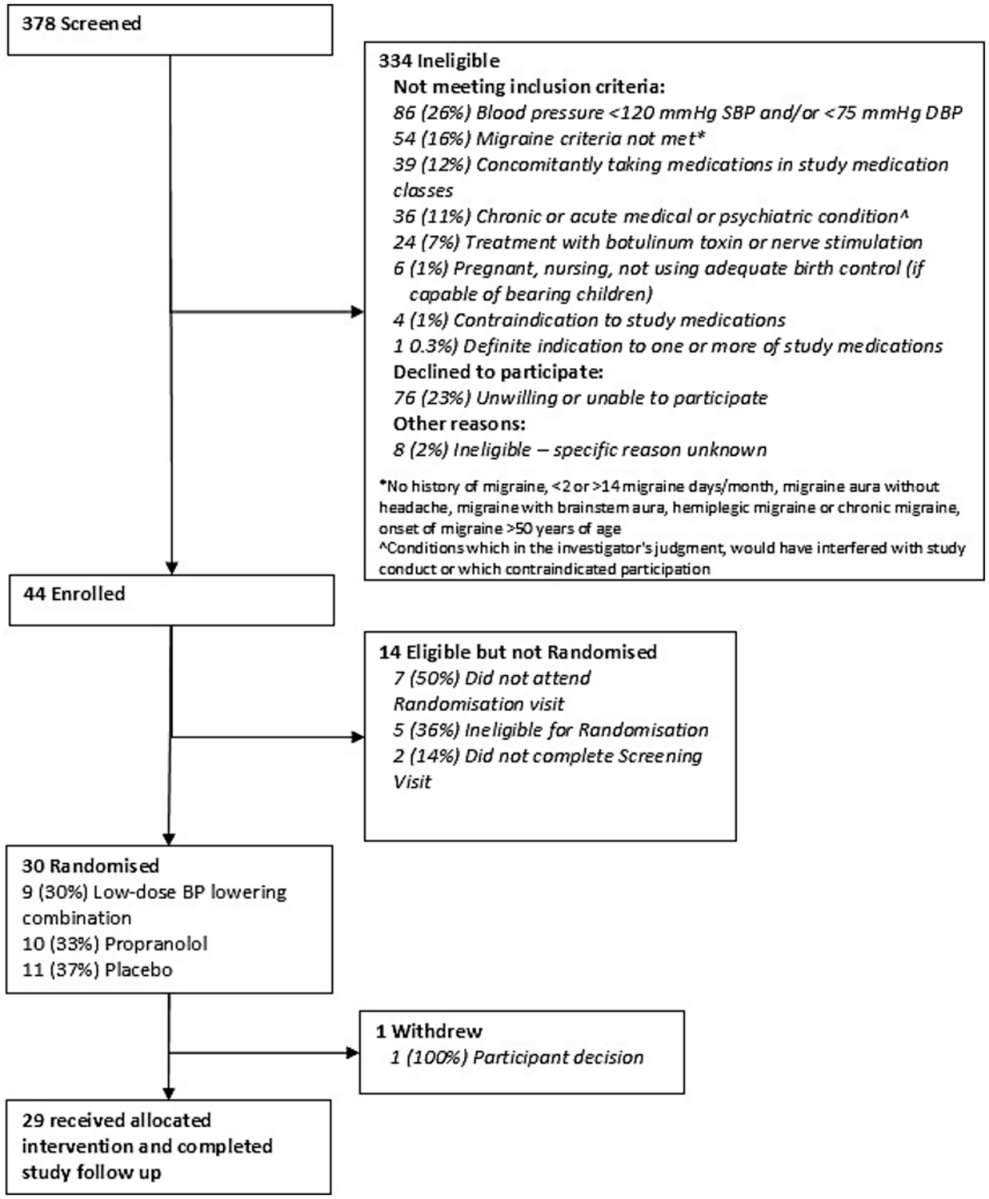
Consort diagram.

Between March 2017 and June 2018, 378 participants were screened, and 30 (8% feasibility) were eligible and randomly allocated to the treatment groups (Figure 2). For the 334 not eligible, 26% had low BP, 23% were unwilling to participate, 16% did not meet episodic migraine criteria and 12% were taking medications that were prohibited during the study. Reasons for unwillingness to participate after initial interest were issues with participating in a drug trial and trial burden. There were 14 participants not randomized after being enrolled because they did not attend randomization visits, were ineligible for randomization due to low BP or deranged blood tests and not completing screening visits. One participant discontinued the study in the first 4 weeks of the trial for personal reasons.

### Baseline characteristics of HAPPy

Baseline characteristics are shown on Supplementary Table 2. The mean age (SD) of the participants was 49 (11.5), 83% were female, 90% Caucasians and 10% Asians and 50% worked full time. The mean body mass index was 27 kg/m^2^, the mean (SD) SBP was 131 (12) mmHg and the mean diastolic BP was 87 (8) mmHg. 16% reported a history of depression. Participants had an average of 67 h of migraine per month (median 67, interquartile range 15–80); 47% reported having aura symptoms, all participants experienced photophobia and over 80% were nauseated. Triptans were the most used abortive therapy and 23% of the participants were on prophylactic medications.

### Outcomes per groups

The low-dose BP treatment combination had on average 0.6 fewer monthly migraine days with wide confidence intervals (95% confidence interval [CI] −2.9 to 1.8) while in the propranolol group there were −1.1 fewer days (95% CI −3.8 to 1.6) (Supplementary Table 2). The effects on BP compared to placebo at 12 weeks had wide confidence intervals, e.g., −7 (−19 to 5) and −8 (−17 to 0) mm Hg systolic reductions for low-dose combination and propranolol, respectively, (Table 1). There was no significant difference in any other outcomes. There were no significant differences in outcomes in any of the pre-defined subgroups, including by age or sex (Supplementary Table 3).

**Table 1:** Effects of treatment on migraine related outcomes and blood pressure.

Outcomes	Change from baseline to week 12			Treatment effects compared to placebo (95% CI)*	
	Low-dose blood pressure lowering combination (*N* = 9)	Propranolol (*N* = 10)	Placebo (*N* = 11)	Low-dose blood pressure lowering combination vs. placebo	Propranolol vs. placebo
Change in monthly headache days, Mean (SD)	−2.9 (2.2)	−3.4 (3)	−2.3 (2.7)	−0.6 (−2.9, 1.8)	−1.1 (−3.8, 1.6)
Change in monthly hours with headache, Mean (SD)	−21.2 (20.6)	−26.6 (32.1)	−13.7 (24.0)	−7.5 (−34.1, 19.3)	−12.9 (−45.5, 19.7)
Proportion of subjects with ≥ 50% reduction in monthly headache, n (%)	6/9 (66%)	8/10 (80%)	5/10 (50%)	1.3 (0.6, 2.9)	1.6 (0.8, 3.2)
Change in monthly headache medication treatment days, Mean (SD)	−2.2 (2.4)	−3.6 (4.5)	−2.5 (2.9)	0.3 (−2.8, 3.5)	−1.1 (−5.6, 3.4)
Change in systolic blood pressure, Mean (SD)	−9.6 (16.5)	−10.9 (10.1)	−2.5 (7.7)	−7.1 (−19.3, 5.2)	−8.4 (−16.8, −0.0)
Change in diastolic blood pressure, Mean (SD)	−5.1 (9.1)	−10.5 (6.4)	−3.0 (6.6)	−2.2 (−9.8, 5.5)	−7.5 (−13.6, −1.3)

*All treatment effects are mean difference, except 50% reduction in monthly headache which is relative risk.

### Adverse events

AEs are shown in Supplementary Table 4. There were no reported serious AEs. The two most common AEs reported between week 4 and week 12, apart from headache, were feeling faint/dizzy and insomnia. The former was seen in 40% participants of all participants but mostly in the low-dose BP lowering combination arm (78%) and the placebo arm (64%). There were a similar percentage of participants reporting insomnia in all groups (between 33 and 55%). There were no dropouts due to adverse events.

## Discussion

The findings from our pilot study can be used to inform the design and conduct of BP lowering trials in migraine—in particular regarding the need to screen large numbers of participants. There was adequate interest during recruitment through the community via traditional and social media and in outpatient clinics, however a more specific recruitment strategy would be needed to avoid a screen failure rate of over 90%. A majority of the participants were women who were Caucasian and in their late forties; we therefore learnt that recruitment of a more heterogeneous sample in BP trials for the treatment of migraine warrants consideration in future research. Nevertheless, we found that the low-dose BP lowering combination pill was tolerable and safe in this population.

Based on two previous systematic reviews, evaluating headache in general, but not migraine specifically, all classes of antihypertensive medications showed effectiveness in reducing headache compared to placebo, suggesting that lowering BP per se may play a key role in this effect (2, 5, 6). Other hypothesized mechanisms of these medications in migraine prophylaxis include: reduction in the hyperactivity of renin-angiotensin-aldosterone, catecholaminergic and adrenergic systems; inhibition of excessive vasoconstriction due to 5-HT release; inhibition of neurogenic inflammation; regulation of central neuronal activity; membrane stabilization; and modulation of serotonin (7).

The main limitation of our trial was the small sample size, although it was only a pilot study. Hence this study was not powered to assess efficacy; rather, it was designed to assess feasibility, safety, and tolerability. The imprecision in the treatment effect estimates—reflected in wide confidence intervals—is expected in this context and highlights the need for larger trials to reliably assess efficacy. A major barrier to recruitment was identifying individuals with BP above 120/75 mmHg. While this threshold was intended to mitigate the risk of hypotension with a triple antihypertensive combination, it may have excluded a substantial proportion of otherwise eligible individuals with migraine. For future trials, a broader inclusion range, consideration of dose titration, or recruitment from populations with prior elevated BP (e.g., those with a history of hypertension or hypertensive disorders of pregnancy) may improve feasibility while maintaining participant safety. Although the association between BP levels and migraine is controversial, some population-based data have found that people with migraine have lower baseline SBP compared to those without migraine (8). In addition, our recruitment identified many individuals who were unwilling to participate after discovering that the trial was a drug trial. Study co-design with people with lived experience of migraine, especially on recruitment strategy and trial procedures would likely improve gender and ethnic diversity and participation in the study.

Migraine, in particular migraine with aura, increases the risk of stroke and other types of cardiovascular disease (9). Antihypertensive therapy reduces risk of stroke, coronary heart disease, and congestive heart failure in a wide range of patients, largely irrespective of BP level, and the degree of BP reduction is an important determinant of the degree of cardiovascular risk reduction (10–13). Further research is required on comparative efficacy of BP lowering strategies that are more tolerable than existing medications such as propranolol; and to determine whether greater BP lowering leads to more benefits in migraine prevention (2).

## Conclusion

This pilot study showed the tolerability and safety of a low-dose triple combination BP lowering medication in the treatment of episodic migraine, providing the rationale for a larger trial to assess efficacy.

## Data Availability

The de-identified data will be made available upon reasonable request to the corresponding author.
