# Supplementary material for: Blood pressure lowering for prevention of episodic migraine: results of a pilot randomized, placebo-controlled trial of combination blood pressure lowering medication with propranolol
Source: Front Neurol. 2025 Sep 16;16:1630732. doi: 10.3389/fneur.2025.1630732 (PMC12479289; doi:10.3389/fneur.2025.1630732)
Supplement: Supplementary file 1 [file Data_Sheet_1.docx]

Supplementary Tables

Supplemental Table 1: Eligibility Criteria for HAPPy trial

| Inclusion Criteria | Exclusion criteria |
| --- | --- |
| - Migraine headache (with or without typical aura) according to the diagnostic criteria of the International Headache Society - 2-14 days per month with migraine headache averaged over past 3 months (90-days), as self-reported by subject - Migraine symptoms must have been present for ≥1 year prior to enrolment in the study. - Onset of migraine symptoms must have occurred before the age of 50 years - Adults between 18 and 65 years - Office SBP ≥120mmHg and/or DBP ≥75 mmHg - No definite contraindications to any of the study medications at the doses used in this trial. (Subjects can be taking other preventive and therapeutic medications as long as they do not contraindicate study medication. Patients will not be eligible if they are taking medications from the same class as the study treatments) - Is medically stable as determined by the Study Investigator - If taking any concomitant migraine preventative medication(s), is on a stabilised dosage at the discretion of the Investigator - Is willing to stay on current migraine preventative medication(s) for the duration of the study - Is able to take oral medication, adhere to the medication regimens, and perform study procedures over the study duration | - Contraindication to any of telmisartan, amlodipine, indapamide, rosuvastatin, ezetimibe, simvastatin or propranolol - Concomitantly taking an angiotensin receptor blocker, angiotensin converting enzyme inhibitor, calcium channel blocker, diuretic or statin. - Definite indication to any one or more of the study medications - Subject has history of cluster headaches - Subject who exclusively has migraine aura without headache, migraine with brainstem aura, hemiplegic migraine or chronic migraine - Medication overuse headaches according to International Headache Society criteria - Female patients who are pregnant, nursing, or those not using adequate birth control, if capable of bearing children. - Chronic medical illnesses (e.g. lupus) that could potentially affect frequency of headache as determined by the Study Investigator - Alcohol or substance abuse within the last year - Any concurrent medical or psychiatric condition which, in the investigator's judgment, may interfere with study conduct or which contraindicates participation - Abnormal creatinine or electrolytes on screening. - Inability to provide informed consent. - Treatment for migraine with botulinum toxin injections or nerve stimulation in the past three months - Participation in an interventional medical investigation or clinical trial currently or within the past three months. Subjects in observational, natural history and/or epidemiological studies not involving an intervention are eligible |

Supplemental Table 2 Baseline Characteristics of trial participants

| Baseline Characteristics | Low-dose blood pressure lowering combination (N=9) | Propranolol (N=10) | Placebo (N=11) | Overall (n=30) |
| --- | --- | --- | --- | --- |
| Age (years), mean (SD) | 47 (12) | 51 (10) | 46 (12) | 48 (12) |
| Gender (female), n (%) | 8 (89%) | 8 (80%) | 9 (82%) | 25 (83%) |
| Ethnicity (Caucasian), n (%) | 8 (89%) | 8 (80%) | 11 (100%) | 27 (90%) |
| Highest Educational Qualification. n (%)   - School and diploma - University | 4 (44%)  5 (56%) | 6 (60%)  4 (40%) | 4 (36%)  7 (64%) | 14 (47%)  16 (53%) |
| Type of employment in the past 3 months, n (%)   - Not employed - Causal - Part time - Full time | 1 (11%)  3 (33%)  3 (33%)  2 (22%) | 1 (10%)  0 (0%)  3 (30%)  6 (60%) | 0 (0%)  1 (9%)  3 (27%)  7 (64%) | 2 (7%)  4 (13%)  9 (30%)  15 (50%) |
| Systolic / diastolic blood pressure (mmHg), mean (SD) | 129 (14) / 87 (7) | 136 (13) / 87 (10) | 128 (8) / 85 (6) | 131 (12) / 87 (8) |
| Smoking n (%)   - Current smoker - Ex-smoker - Non-smoker | 0 (0%)  1 (11%)  8 (89%) | 0 (0%)  3 (30%)  7 (70%) | 1 (9%)  4 (36%)  6 (55%) | 1 (3%)  8 (27%)  21 (70%) |
| Migraine headache frequency (days per month), mean (SD) | 5 (2) | 6 (3) | 5 (2) | 5 (3) |
| Acute symptomatic migraine treatment, n (%)   - Triptans - Non-steroidal anti-inflammatory drugs (NSAIDs) - Ergotamines - Other | 6 (67%)  3 (33%)  0 (0%)  3 (33%) | 7 (70%)  3 (30%)  0 (0%)  6 (60%) | 6 (55%)  5 (46%)  0 (0%)  6 (50%) | 19 (63%)  11 (37%)  0 (0%)  15 (55%) |
| Current preventative treatment, n (%)   - Amitriptyline - Pizotifen - Topiramate - Beta-blocker - Other | 0 (0%)  1 (11%)  0 (0%)  0 (0%)  1 (11%) | 0 (0%)  0 (0%)  0 (0%)  0 (0%)  2 (20%) | 1 (9%)  0 (0%)  1 (9%)  0 (0%)  1 (9%) | 1 (3%)  1 (3%)  1 (3%)  0 (0%)  4 (13%) |

Supplemental Table 3: Subgroup analysis

|  | Groups | | | Overall |
| --- | --- | --- | --- | --- |
| Outcomes (Week 12 vs Baseline) | Low-dose BP lowering combination (N=9) | Propranolol (N=10) | Placebo (N=11) | (N=30) |
| Change in monthly headache days |  |  |  |  |
| Male |  |  |  |  |
| N Mean (SD) | 1 1.0 (-NA-) | 2 -7.0 (4.24) | 2 -1.5 (2.12) | 5 -3.2 (4.32) |
| Female |  |  |  |  |
| N Mean (SD) | 8 -3.4 (1.69) | 8 -2.5 (2.07) | 8 -2.5 (2.88) | 24 -2.8 (2.21) |
| Change in monthly headache days |  |  |  |  |
| Age <= mean |  |  |  |  |
| N Mean (SD) | 4 -3.0 (2.31) | 4 -1.8 (2.22) | 7 -1.9 (1.35) | 15 -2.1 (1.81) |
| Age > mean |  |  |  |  |
| N Mean (SD) | 5 -2.8 (2.28) | 6 -4.5 (3.08) | 3 -3.3 (4.93) | 14 -3.6 (3.10) |
| Change in monthly headache days |  |  |  |  |
| SBP <= mean |  |  |  |  |
| N Mean (SD) | 6 -3.3 (1.97) | 5 -2.0 (1.87) | 8 -2.6 (2.92) | 19 -2.7 (2.33) |
| SBP > mean |  |  |  |  |
| N Mean (SD) | 3 -2.0 (2.65) | 5 -4.8 (3.42) | 2 -1.0 (0.00) | 10 -3.2 (3.12) |
| Change in monthly headache days |  |  |  |  |
| DBP <= mean |  |  |  |  |
| N Mean (SD) | 5 -3.6 (1.67) | 6 -2.2 (1.72) | 9 -2.4 (2.79) | 20 -2.7 (2.23) |
| DBP > mean |  |  |  |  |
| N Mean (SD) | 4 -2.0 (2.58) | 4 -5.3 (3.77) | 1 -1.0 (-NA-) | 9 -3.3 (3.35) |
| Change in monthly headache days |  |  |  |  |
| LDL <= mean |  |  |  |  |
| N Mean (SD) | 6 -2.2 (2.23) | 7 -3.6 (3.60) | 5 -3.8 (3.11) | 18 -3.2 (2.98) |
| LDL > mean |  |  |  |  |
| N Mean (SD) | 3 -4.3 (1.15) | 3 -3.0 (1.00) | 5 -0.8 (0.84) | 11 -2.4 (1.80) |

Supplemental Table 4. Adverse events reported in different study groups

| Adverse events | Groups | | |
| --- | --- | --- | --- |
|  | Low-dose blood pressure lowering combination (N=9) | Propranolol (N=10) | Placebo (N=11) |
| ***Any SAE, n (%)*** | 0 (0%) | 0 (0%) | 0 (0%) |
| ***Adverse events*** |  |  |  |
| Feeling faint/dizziness | 7 (78%) | 3 (30%) | 2 (18%) |
| Syncope | 2 (22%) | 1 (10%) | 0 (0%) |
| Falls | 1 (11%) | 0 (0%) | 0 (0%) |
| Pedal oedema | 1 (11%) | 0 (0%) | 0 (0%) |
| Headache | 9 (100%) | 9 (90%) | 11 (100%) |
| Muscle cramps | 4 (44%) | 3 (30%) | 3 (27%) |
| Bradycardia | 0 (0%) | 1 (10%) | 0 (0%) |
| Flushing | 3 (33%) | 0 (0%) | 2 (18%) |
| Skin rash/itching | 3 (33%) | 3 (30%) | 0 (0%) |
| Gastrointestinal complaints | 3 (33%) | 3 (30%) | 4 (36%) |
| Myalgia | 2 (22%) | 2 (20%) | 3 (27%) |
| Gout | 0 (0%) | 0 (0%) | 0 (0%) |
| Insomnia | 3 (33%) | 4 (40%) | 5 (46%) |
| Nightmares | 1 (11%) | 1 (10%) | 3 (27%) |
